# NK Cell Subgroups, Phenotype, and Functions After Autologous Stem Cell Transplantation

**DOI:** 10.3389/fimmu.2015.00583

**Published:** 2015-11-24

**Authors:** Benedikt Jacobs, Sara Tognarelli, Kerstin Poller, Peter Bader, Andreas Mackensen, Evelyn Ullrich

**Affiliations:** ^1^Department of Cancer Immunology, Institute for Cancer Research, Oslo University Hospital, Radiumhospital, Oslo, Norway; ^2^The KG Jebsen Center for Cancer Immunotherapy, Institute of Clinical Medicine, University of Oslo, Oslo, Norway; ^3^Department of Haematology and Oncology, University Hospital Erlangen, Erlangen, Germany; ^4^Department of Pediatric Stem Cell Transplantation and Immunology, Children’s Hospital, Johann Wolfgang Goethe-University, Frankfurt, Germany; ^5^LOEWE Center for Cell and Gene Therapy, Johann Wolfgang Goethe-University, Frankfurt, Germany

**Keywords:** NK cells, CD57, KIR, autologous stem cell transplantation, CD107a expression, IFN-γ production

## Abstract

High-dose chemotherapy with consecutive autologous stem cell transplantation (autoSCT) is a well-established treatment option for patients suffering from malignant lymphoma or multiple myeloma. Natural killer (NK) cells are an important part of the immune surveillance, and their cell number after autoSCT is predictive for progression-free and overall survival. To improve knowledge about the role of NK cells after autoSCT, we investigated different NK cell subgroups, their phenotype, and their functions in patients treated with autoSCT. Directly after leukocyte regeneration (>1000 leukocytes/μl) following autoSCT, CD56^++^ NK cells were the major NK cell subset. Surprisingly, these cells showed unusually high surface expression levels of CD57 and killer Ig-like receptors (KIRs) compared to expression levels before or at later time points after autoSCT. Moreover, these NK cells strongly upregulated KIR2DL2/3/S2 and KIR3DL1, whereas KIR2DL1/S1 remained constant, indicating that this cell population arose from more immature NK cells instead of from activated mature ones. Remarkably, NK cells were already able to degranulate and produce IFN-γ and MIP-1β upon tumor interaction early after leukocyte regeneration. In conclusion, we describe an unusual upregulation of CD57 and KIRs on CD56^++^ NK cells shortly after autoSCT. Importantly, these NK cells were functionally competent upon tumor interaction at this early time point.

## Introduction

Natural killer (NK) cells are an important part of the innate immune system and are able to kill virus-infected or malignantly transformed cells (1). Their important role in tumor surveillance has been demonstrated in many different tumor models (1). NK cell cytotoxicity is regulated by a diverse repertoire of inhibitory and activating receptors. Inhibitory receptors, such as killer Ig-like receptors (KIRs) and the C-type lectin-like receptor NKG2A, recognize different alleles of HLA molecules (HLA-A, B, and C by KIRs and HLA-E by NKG2A) on healthy cells. In contrast, many tumor cells downregulate their HLA molecules to evade T cell recognition, making them more susceptible to NK cell killing (2). Additionally, tumor cells may express stress-induced molecules, such as MHC I chain-related molecule A/B or UL-16-binding proteins, which are ligands for the activating NK cell receptor NKG2D (3, 4).

High-dose chemotherapy (HDC) with consecutive autologous stem cell transplantation (autoSCT) is an effective and well-established treatment option for patients suffering from multiple myeloma (MM) (5) or malignant lymphoma (6–9). Before treatment with the myeloablative chemotherapy, hematopoietic stem cells are collected from peripheral blood and frozen. Following HDC, these cells are thawed and given back to the patient in order to shorten the time of aplasia, thereby reducing the infection and blood transfusion rates.

Many reports have demonstrated the important role of the absolute lymphocyte count after HDC/autoSCT (10). It has been shown that an absolute lymphocyte count >500/μl is associated with improved overall and progression-free survival in patients with Hodgkin lymphoma (11), non-Hodgkin lymphoma (NHL) (12), acute myeloid leukemia (13), MM (12), and metastatic breast cancer (14). By analyzing the different lymphocyte subsets at day 15 following autoSCT, a clear correlation between improved overall survival and progression-free survival could only be found for NK cell counts >80/μl. No correlation was found for any other lymphocyte subset (15). In a more recent study, improved median overall and progression-free survival as well as the NK cell count at day 15 after HDC/autoSCT were all associated with an increased IL-15 concentration at day 15 of ≥76.5 pg/ml for NHL patients receiving HDC/autoSCT (16).

Because there is no information available regarding the detailed analysis of NK cell subsets or function early after HDC/autoSCT, in our study, we prospectively investigated the major NK cell subsets directly after leukocyte recovery (leukocytes >1000/μl) and also at later time points after HDC/autoSCT in patients with different lymphoproliferative diseases. Moreover, we further analyzed the different NK cell subsets, evaluating their education and differentiation markers, as well as their functional properties, such as cytokine/chemokine production and degranulation capacity.

## Materials and Methods

### Patients’ Characteristics and Study Design

This study was carried out in accordance with the recommendations of the local ethics committee of the University of Erlangen, and all patients gave written informed consent in accordance with the Declaration of Helsinki. Patients who suffered from MM or malignant lymphoma and received HDC/autoSCT were included. Blood was taken from these patients at three different time points. Time point 1 (TP1) was before the start of the HDC and at least 3 weeks after the last chemotherapy. The second time point (TP2) was 1–2 days after leukocyte regeneration (>1000 leukocytes/μl) following autoSCT, and the third time point (TP3) was after at least 2 weeks following leukocyte recovery.

### Reagents

For NK and K562 cell culture, we used full media containing RPMI 1640 media (Gibco^®^) supplemented with 10% FBS, MEM non-essential amino acids (1%), sodium pyruvate (1%), l-glutamine (1%; all from PanBiotech), and penicillin/streptomycin (1%; Thermo Fischer Scientific). For the washing steps, we used Dulbecco’s phosphate-buffered saline (DPBS; Gibco^®^).

To analyze the different leukocyte subsets, CD3, CD14, CD16, CD19, CD45, and CD56 antibodies with different fluorochromes from Becton Dickinson (BD) were used. For detailed NK cell subset analyses, we used anti-KIR2D-, KIR3DL1/2-, and KIR2DL1/S1-PE (Miltenyi), KIR2DL1-PerCP (R&D), KIR2DL2/3/S2-APC (Beckman Coulter), KIR3DL1 PE-Vio770 (Miltenyi), NKG2A FITC (Miltenyi), and CD57 APC (BD). For the KIR staining, the clones of the antibodies were selected according to Czaja et al. (17), and a sequential staining protocol was used as described by Beziat et al. (18). For intracellular staining, we used IFN-γ PE-Cy-7 and MIP-1β APC-H7 (BD). To exclude dead cells, 7-AAD (BD) for extracellular and Fixable Viability Dye eFluor^®^ 520 (eBioscience) for intracellular staining were used.

### PBMC Preparation, Freezing, and Thawing

Blood samples were obtained from the patients at the indicated time points. PBMCs were isolated by performing a Ficoll density centrifugation of whole blood samples, and then the PBMCs were frozen (5 × 10^6^ PBMCs/ml freezing media containing 90% FCS + 10% DMSO; from Sigma) until they were used.

For thawing, tubes were incubated at room temperature and gently thawed by re-suspending the cells in prewarmed full media (without FCS). The cells were washed twice and counted before being used for further analysis.

### Extra- and Intracellular Antibody Staining

For surface staining, the cells were incubated with different antibody cocktails for 10′ at 4°C; then, they were washed and either fixed in BD CytoFix™ solution or further processed for intracellular staining using the BD Cytofix/Cytoperm™ kit. Briefly, the cells were first incubated for 30′ at 4°C with Fixable Viability Dye eFluor^®^ 520 (eBioscience), and then they were washed and fixed for 20′ at 4°C in 100 μl BD Cytofix™ solution. Subsequently, the cells were washed in BD Cytoperm™ solution and incubated with the indicated intracellular antibodies for 20′ at 4°C. Finally, the cells were washed, placed into BD CytoFix™ solution, and analyzed with a BD FACSCanto II™ or Canto10c™ using the FlowJo^®^ software (FlowJo, LLC) was used to analyze the FACS data.

### Functional assays

After thawing, the cells were placed into full media supplemented with 100 IU IL-2/ml (Proleukin^®^, Novartis) in a 96-U well plate overnight. The cells were harvested, washed, counted, and incubated with K562 cells (ratio 1:1) in full media in a 96-U well plate for 4 h. CD107a APC (BD) was added at the start of the coculturing period and BD GolgiStop™ (BD) was added after 1 h for the rest of the incubation time.

### Statistics

For statistical analysis, we used GraphPad Prism^®^ software. In all graphs, the mean and SD were calculated and plotted. For comparison between matched samples, we used a Wilcoxon test, whereas for non-matched samples, we performed a Mann–Whitney test. Statistical significance is indicated with the *p*-values (*<0.05; **<0.01; ***<0.001; *****<0.0001).

## Results

### Patients’ Characteristics and Leukocyte Subsets

Peripheral blood samples from 32 different patients collected at three specific time points (TP1–3, as described in Section “Materials and Methods”) were available for the analysis of leukocyte subsets. The basic patient characteristics are summarized in Table 1. The ratio between male and female patients was approximately 2:1. Half of the patients suffered from MM. The average age at HDC/autoSCT was 56.7 years. The average time between SCT and TP2 was 11.6 days, whereas the time between collecting samples at TP2 and TP3 was 38.8 days. All three values were normally distributed.

**Table 1:** **Patient characteristics**.

**Gender**	Male (23), female (9)
**Mean age (range)**	56.7 years (30–74 years)
**Malignancy**	
Multiple myeloma	16
Diffuse large-cell B cell lymphoma	7
Mantle cell lymphoma	3
T cell lymphoma	5
Hodgkin lymphoma	1
**Recurrent/refractory disease at 1 year after SCT (yes/no)**	6/21 (5 patients were lost during follow-up)
**Time period SCT–TP2 (mean, days)**	11.6
**Time period TP2–TP3 (mean, days)**	38.8

**Figure 1:**
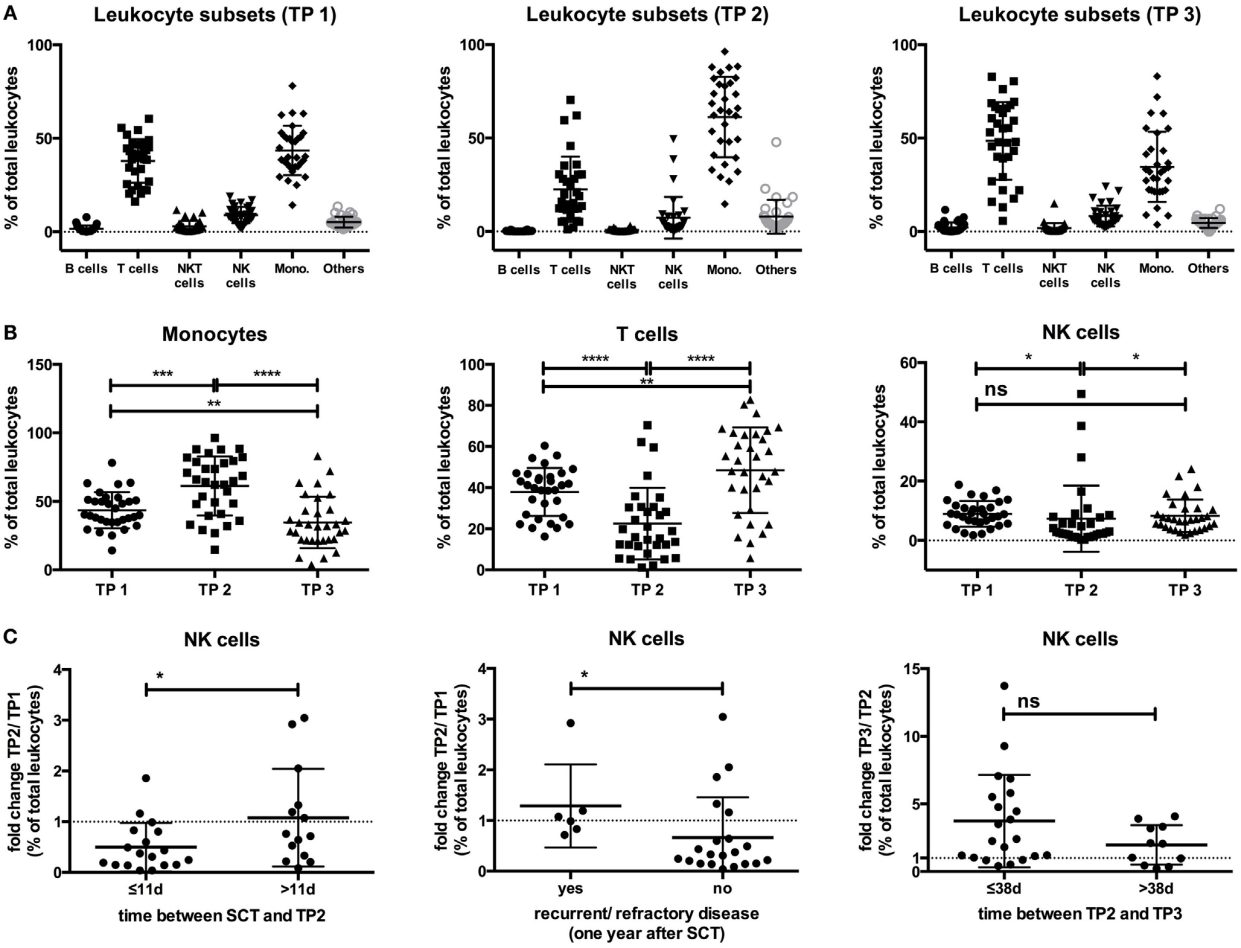
**(A)** The percentages of the different leukocyte subsets within the leukocyte population at all three time points are indicated. **(B)** The percentage of CD14^+^ monocytes within the leukocyte population increased from TP1 (43.49%) to TP2 (61.22%; *p*-value: 0.0003), becoming the major leukocyte subset at TP2 but decreased below the starting levels at TP3 (34.6%; *p*-value TP2/TP3: <0.0001; TP1/3: 0.0088). In contrast, the T cell percentages decreased from TP1 (37.89%) to TP2 (22.5%; *p*-value: <0.0001), but they increased again at TP3 (48.49%) above the initial values to become the major leukocyte subset at TP3 (*p*-value TP2/TP3: <0.0001; TP1/3: 0.0093). The NK cell percentages decreased from TP1 (8.94%) to TP2 (7.32%) but reached the initial levels again at TP3 (8.29%). **(C)** Patients with a time period of ≤11 days between SCT and TP2 had a decrease from TP1 to TP2 within their NK cell percentage (ratio TP2/TP1: 0.49), in contrast to patients with a time period >11 days (ratio TP2/TP1: 1.07). Similarly, patients with no recurrent/refractory disease 1 year after SCT had a decrease of their NK cell percentage at TP2 (ratio TP2/TP1: 0.66), as opposed to patients who were recurrent/refractory at 1 year (ratio TP2/TP1: 1.28). The increase of the NK cell percentage from TP2 to TP3 was more pronounced in patients with a time period of ≤38 days between TP2 and TP3 (ratio TP3/TP2: 3.73) than in patients with a time period of >38 days (ratio TP3/TP2: 1.97).

Before the start of HDC/autoSCT at TP1, CD3^+^CD56^−^ T cells and CD14^+^ monocytes were the two major leukocyte subsets in all patients (Figure 1A; for gating strategy, see Figure S1 in Supplementary Material). While CD14^+^ monocytes were the major subset early after leukocyte recovery after HDC/autoSCT at TP2 (Figures 1A,B), CD3^+^CD56^−^ T cells were the major subset at TP3 (Figures 1A,B). In contrast, the NK cell percentages within the leukocyte population significantly decreased from TP1 to TP2 (*p*-value: 0.0175) but recovered to the initial value at TP3 (*p*-value TP2/TP3: 0.0263; TP1/3: 0.19; Figures 1A,B).

By correlating the NK cell dynamics at the three different time points with clinical data, we observed that the fold change of the NK cell percentage within the leukocyte population between TP1 and TP2 (ratio TP2/TP1) significantly differed between patients having a time period of ≤11 days between SCT and TP2 and those having a period of >11 days (*p*-value: 0.04). When the time period was >11 days, no decrease in the NK cell percentage within the leukocyte population at TP2 was observed. Moreover, another significant difference was observed when comparing the fold change in the NK cell percentage between TP1 and TP2 in patients who were refractory/recurrent or not at 1 year after SCT (*p*-value: 0.0258). Patients with recurrent or refractory disease did not have a decrease in their fold change ratio (TP2/TP1), while patients without recurrent/refractory disease did have a decrease at 1 year after SCT. Additionally, the fold increase in the NK cell percentage between TP2 and TP3 (TP3/TP2 ratio) was more pronounced when the time period between TP2 and TP3 was ≤38 days (*p*-value: 0.12; Figure 1C).

No differences were observed when analyzing the patients’ age or hematological malignancies in relation to the fold changes of the NK cell percentages within the leukocyte population between the three different time points (Figure S2 in Supplementary Material).

### CD56^++^CD16^−/+^ NK Cells are the Major Subset at Leukocyte Recovery

Next, we analyzed the different NK cell subsets based on their CD56 and CD16 expression. NK cells were divided into CD56^++^CD16^−^ or CD16^+^ and CD56^+^CD16^++^ NK cells (see Figure S1 in Supplementary Material). The CD56^++^CD16^+^ population has been reported to be an intermediate state between CD56^++^CD16^−^ and CD56^+^CD16^++^ NK cells (19, 20). The CD56^+^CD16^−^ population was excluded, as it was shown that this population could be induced by cryopreservation (21).

**Figure 2:**
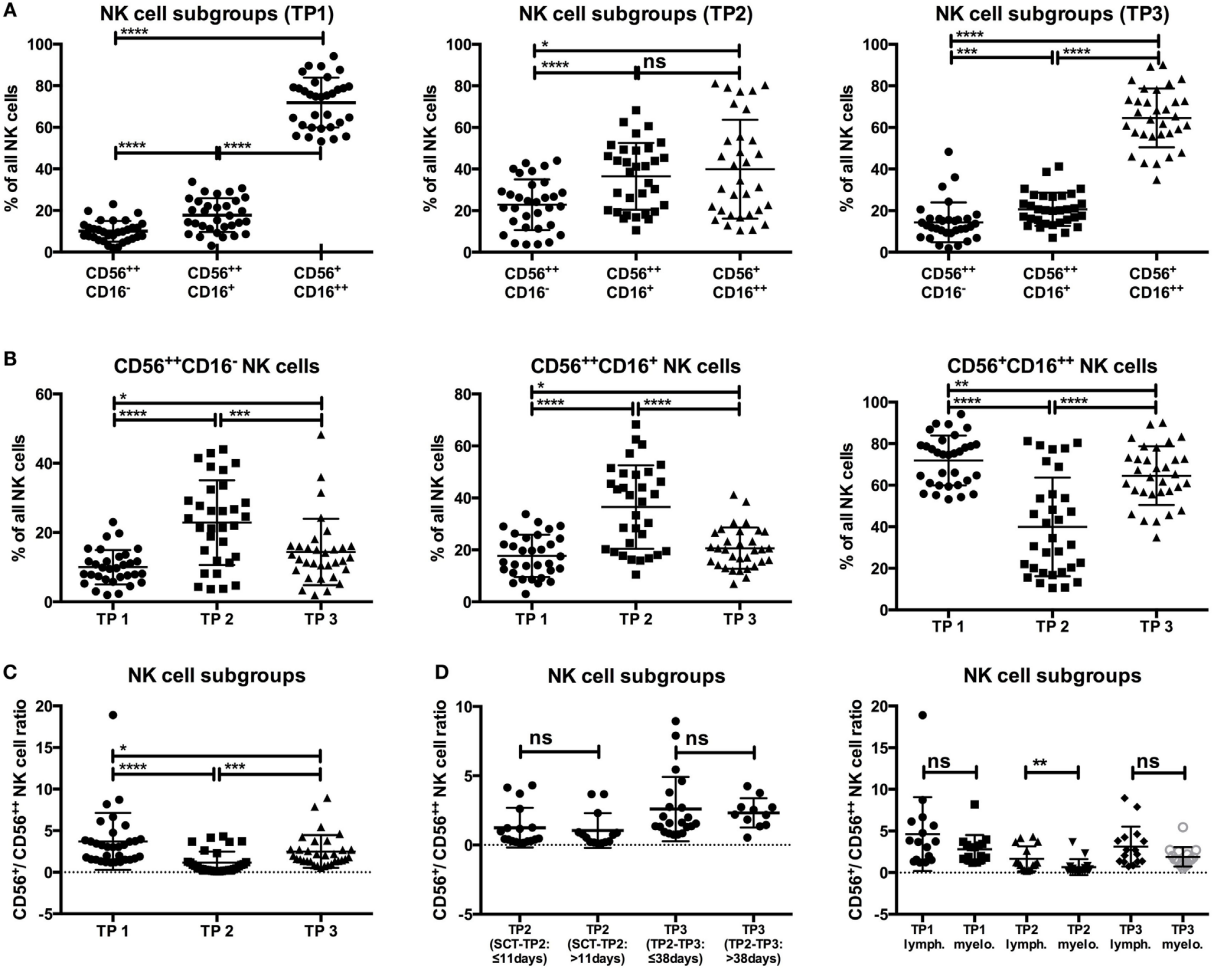
**(A,B)** The level of CD56^+^CD16^+^^+^ NK cells significantly dropped at TP2 (39.98%) and recovered at TP3 (64.58%), but it remained lower in contrast to the starting levels (TP1: 71.86%). Moreover, the percentages of CD56^++^CD16^−/+^ NK cells were markedly increased at TP2 (CD16^−^: 22.85%; CD16^+^: 36.51%) and remained elevated at TP3 (CD16^−^: 14.41%; CD16^+^: 20.66%) compared to the TP1 values (CD16^−^: 10%; CD16^+^: 17.71%). **(C)** The ratio of CD56^+^/CD56^++^ NK cells at TP1 was already lower than in healthy controls (approx 10; data not shown), with a ratio of 3.707, and decreased further at TP2 (1.157). Although the ratio increased at TP3 (2.49), it was still lower compared to TP1 and healthy control samples. **(D)** There were no significant differences when analyzing the CD56^+^/CD56^++^ ratio at TP2 with regard to the time period between SCT and TP2 (≤11 days: 1.247; >11 days: 1.041) or at TP3 with regard to the period between TP2 and TP3 (≤38 days: 2.59; >38 days: 2.32). A significant difference of the CD56^+^/CD56^++^ ratio between lymphoma and myeloma patients was only observed at TP2 (lymphoma patients: 1.656; myeloma patients: 0.6578).

At TP1, the major NK cell subset was the CD56^+^CD16^++^ NK cell population (71.86%), followed by the CD56^++^CD16^+^ (17.71%) and the CD56^++^CD16^−^ (10%) populations (Figure 2A). After leukocyte regeneration (TP2), the CD56^+^CD16^++^ cells significantly decreased (39.98%; *p*-value: <0.0001), whereas both CD56^++^ NK cell subsets significantly increased (CD16^−^: 22.85%, *p*-value: <0.0001; CD16^+^: 36.51%, *p*-value: <0.0001). At TP3, the levels of CD56^+^CD16^++^ NK cells increased again (64.85%; *p*-value: <0.0001) but remained reduced in contrast to the starting levels at TP1 (*p*-value: 0.0064). Conversely, the levels of CD56^++^CD16^−^ (14.41%; *p*-value: 0.0006) and CD16^+^ (20.66%; *p*-value: <0.0001) cells decreased but remained significantly elevated compared to the starting values [*p*-values: 0.0184 (CD16^−^) and 0.0428 (CD16^+^); Figure 2B].

The ratio between mature CD56^+^ and more immature CD56^++^ NK cells, which is approximately 10 within healthy donors (data not shown), was already significantly reduced within our patients before HDC/autoSCT (TP1; ratio: 3.707) because all patients had previously received chemotherapy. Nevertheless, the ratio significantly dropped at TP2 (ratio: 1.157; *p*-value: <0.0001) and did not recover to initial values at TP3 [ratio: 2.49; *p*-value: 0.0001 (TP2–3); 0.0111 (TP1–3); Figure 2C].

The CD56^+^/CD56^++^ ratios at TP2 and TP3 were both independent of the time period between SCT and TP2 (≤11 days: 1.247 vs. >11 days: 1.041; *p*-value: 0.392) and between TP2 and TP3 (≤38 days: 2.59 vs. >38 days: 2.32; *p*-value: 0.504; Figure 2D). Within the group of MM patients, the CD56^+^/CD56^++^ ratio was lower compared to lymphoma patients, but it was only significantly lower at TP2 (1.656 vs. 0.6578; *p*-value: 0.0019; Figure 2D). In contrast, the patients’ age and relapse/refractory status at 1 year after autoSCT seemed to have no impact on the CD56^+^/CD56^++^ ratio at any time point (Figure S3 in Supplementary Material).

### Increased Levels of CD57 and KIR Expression After Leukocyte Regeneration

Next, we analyzed the expression of markers for NK cell education and differentiation at the indicated time points.

**Figure 3:**
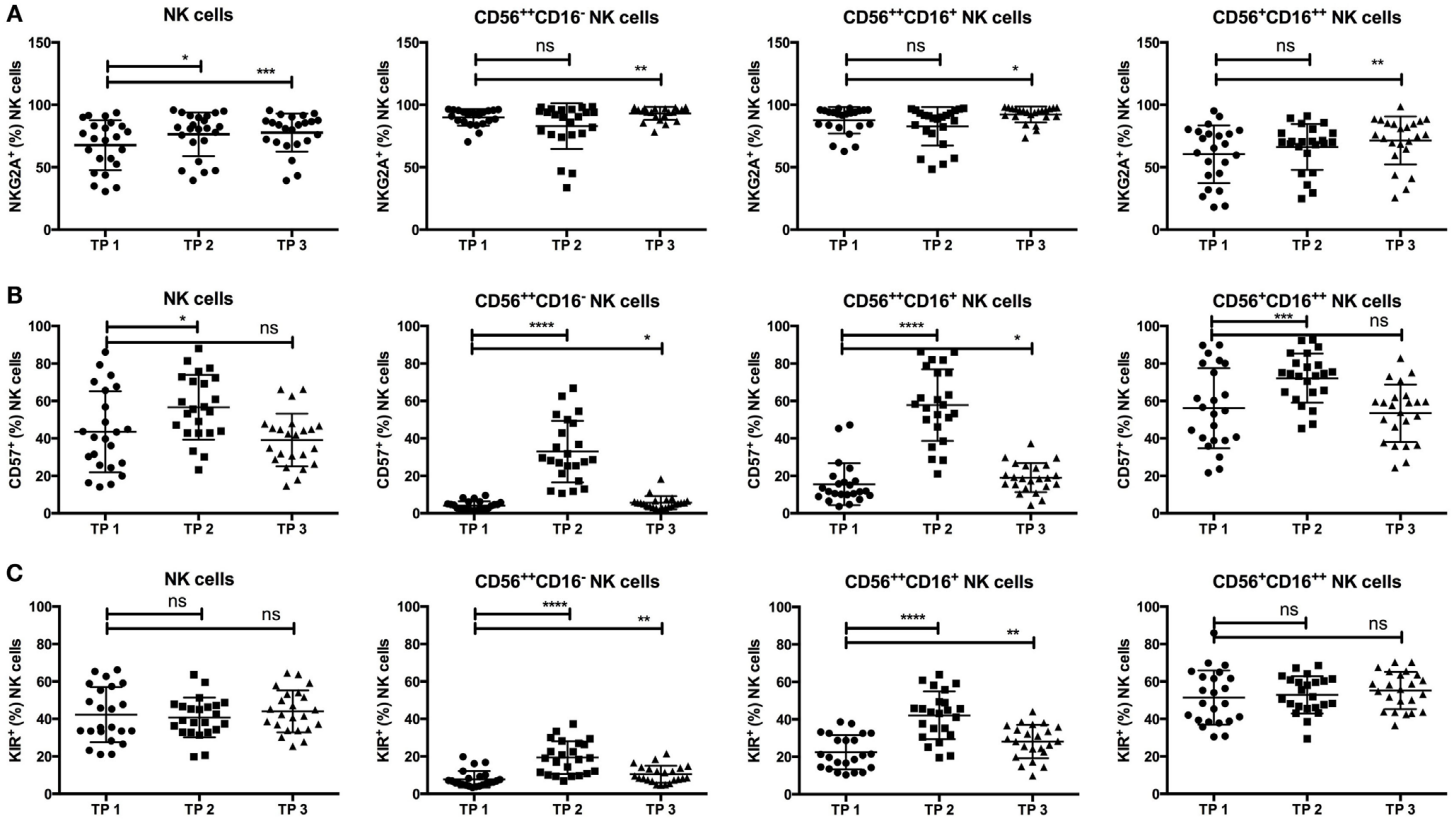
**(A)** NKG2A expression was significantly increased at TP2 (76.41%) and TP3 (77.67%) within all NK cells compared to the starting values (TP1: 67.63). Moreover, the percentage of NKG2A^+^ cells was significantly increased at TP3 within the CD56^++^CD16^−^ (TP1: 89.94%; TP3: 93.1%) and CD16^+^ (TP1: 87.6%; TP3: 92.27%) subsets as well as within the CD56^+^CD16^++^ subset (TP1: 60.39%; TP3: 71.42%). **(B)** Similarly, the percentage of CD57^+^ NK cells increased at TP2 (56.66%) but returned to the initial values at TP3 (TP1: 43.53%; TP3: 39.13%). Consistently, within the CD56^++^CD16^−^ and CD16^+^ as well as within the CD56^+^CD16^++^ NK cell subsets, there was a highly significant increase of CD57 expression at TP2 (CD56^++^CD16^−^: 32.98%; CD56^++^CD16^+^: 57.78%; and CD56^+^CD16^++^: 72.15%), which decreased at TP3 again but remained above the starting values within the CD56^++^ subsets (CD16^−^: TP1 4.1% and TP3 5.592% and CD16^+^: TP1 15.58% and TP3 19.06%). **(C)** In parallel, the KIR expression was significantly elevated at TP2 (CD16^−^: 19.35%; CD16^+^: 42.14%) and TP3 (CD16^−^: 10.43%; CD16^+^: 28.14%) compared to TP1 (CD16^−^: 7.82%; CD16^+^: 22.44%) within the CD56^++^ subsets but not within the CD56^+^CD16^++^ cells (TP1: 51.42%; TP2: 52.89%; and TP3: 55.23%).

As expected, the NKG2A expression on all NK cells increased from TP1 (67.63%) to TP2 (76.51%; *p*-value: 0.0179), and the percentage of NKG2A^+^ NK cells remained elevated above the starting values until TP3 (77.67%; *p*-value: 0.0009). Further NK cell subset analyses revealed that the observed early increase in NKG2A at TP2 was potentially an effect of the elevated CD56^++^ population expressing higher levels of NKG2A than the CD56^+^CD16^++^ population because the NKG2A expression did not significantly differ within the distinct subsets between TP1 and TP2. In contrast, the percentage of NKG2A-expressing NK cells was significantly elevated in all subsets at TP3 compared to TP1 [*p*-value: 0.0054 (CD56^++^CD16^−^); 0.0259 (CD56^++^CD16^+^); 0.0031 (CD56^+^CD16^++^); Figure 3A].

CD57^+^ NK cells, known to define terminally differentiated NK cells, significantly increased from TP1 (43.53%) to TP2 (56.66%; *p*-value: 0.0163) in all NK cells but decreased to the initial values at TP3 (39.13%; *p*-value: 0.4274). Surprisingly, CD57 expression was significantly increased within the CD56^++^CD16^+/−^ population at TP2 (CD16^−^: 32.98%; CD16^+^: 57.78%, *p*-value: <0.0001) but decreased again from TP2 to TP3 (CD16^−^: 5.592%; CD16^+^: 19.06%, *p*-value: <0.0001). Nevertheless, the percentage of CD57^+^ cells was still elevated in contrast to the starting values at TP1 [CD16^−^: 4.1%; CD16^+^: 15.58%, *p*-values: 0.0102 (CD16^−^); 0.0214 (CD16^+^)]. CD57 expression within the CD56^+^CD16^++^ population was also elevated at TP2 (72.15%; *p*-value: 0.0006) but to a much lesser extent (Figure 3B).

Strikingly, the percentage of KIR^+^ NK cells remained constant over time (TP1: 42.45%; TP3: 44.09%; *p*-value: 0.463), even shortly after leukocyte regeneration (TP2: 40.77%; *p*-value: 0.4106). Of note, the KIR expression within the CD56^++^CD16^+/−^ NK cell population was markedly increased at TP2 (CD16^−^: 19.35%; CD16^+^: 42.14%; *p*-value: <0.0001 for both populations) and remained elevated at TP3 (CD16^−^: 10.43%; CD16^+^: 28.14%) in comparison to the starting values at TP1 [CD16^−^: 7.82%; CD16^+^: 22.44%; *p*-values: 0.0083 (CD16^−^); 0.0015 (CD16^+^)]. KIR expression within the CD56^+^CD16^++^ population remained stable throughout all time points (TP1: 51.42%, TP2: 52.89%, and TP3: 55.23%; Figure 3C).

### Elevated CD57 and KIR Expression is Age Dependent

**Figure 4:**
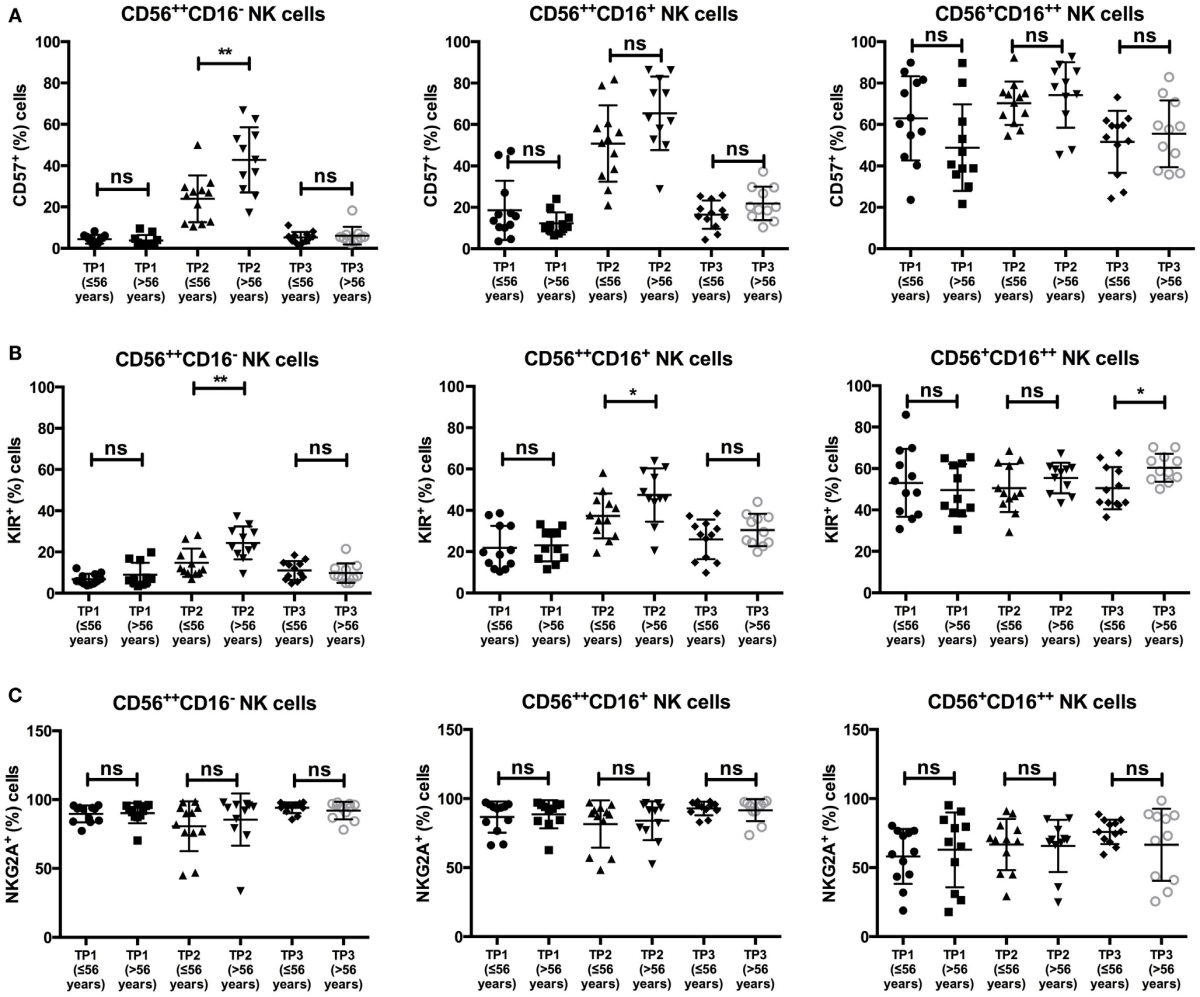
**(A)** There was a significant difference in the average of CD57^+^ cells within the CD56^++^CD16^−^ NK cell subsets at TP2 in patients ≤56 years (23.98%) vs. >56 years (42.8%). A significant difference was not observed within the CD56^++^CD16^+^ (≤56 years: 50.83% vs. >56 years: 65.36%) or the CD56^+^CD16^++^ subset (≤56 years: 70.25% vs. >56 years: 74.23%). **(B)** The percentage of KIR^+^ NK cells at TP2 differed significantly between the two age groups within the CD56^++^ subset (≤56 years: CD56^++^CD16^−^: 14.74% and CD56^++^CD16^+^: 37.29% vs. >56 years: CD56^++^CD16^−^: 24.39% and CD56^++^CD16^+^: 47.44%). A significant difference within the CD56^+^CD16^++^ subset was observed at TP3 (≤56 years: 50.51% vs. >56 years: 60.37%). **(C)** No difference was observed in NKG2A expression within the different NK subsets and age groups.

Additionally, we analyzed the influence of different clinical factors on the expression levels of NKG2A, CD57, and KIRs within the different NK cell subsets. As CD57 expression levels within healthy individuals are known to be age dependent (22), we compared CD57 expression within the younger (≤56 years) and older (>56 years) patient populations. Notably, there was a significant difference in the percentage of CD57^+^ cells within the CD56^++^CD16^−^ population at TP2 (≤56 years: 23.98%; >56 years: 42.8%; *p*-value: 0.0056), whereas this effect was not observed within the CD56^++^CD16^+^ or the CD56^+^CD16^++^ population (Figure 4A).

Similar, by analyzing KIR expression within the CD56^++^CD16^+/−^ populations, we observed a significant difference in their expression at TP2 between the two age groups [≤56 years: 14.74% (CD16^−^) and 37.29% (CD16^+^); >56 years: 24.39% (CD16^−^) and 47.44% (CD16^+^); *p*-values: 0.0088 (CD16^−^), 0.0317 (CD16^+^)]. No age-dependent difference was found for KIR expression within the CD56^+^CD16^++^ population at TP2 but at TP3 (≤56 years: 50.51%; >56 years: 60.37%; *p*-value: 0.0225; Figure 4B).

In contrast, there was no difference in NKG2A expression throughout all age groups, time points, or NK cell populations (Figure 4C).

We also addressed CD57, KIR, and NKG2A expressions in different subsets with regard to recurrent/refractory disease at 1 year after SCT and hematological malignancy. There were no differences in the expression of the three markers regarding the rate of relapsed/refractory disease throughout all NK cell subsets and time points, except for CD57 expression at TP1 within the CD56^++^CD16^−^ NK cell population, which was higher in patients with no recurrent/refractory disease 1 year after SCT (*p*-value: 0.0083; Figure S4A in Supplementary Material).

Furthermore, patients with MM had higher NKG2A expression within the CD56^++^CD16^−/+^ subset at TP2 compared to lymphoma patients. No further differences were observed (Figure S4B in Supplementary Material).

### Detailed KIR Expression Analysis

**Figure 5:**
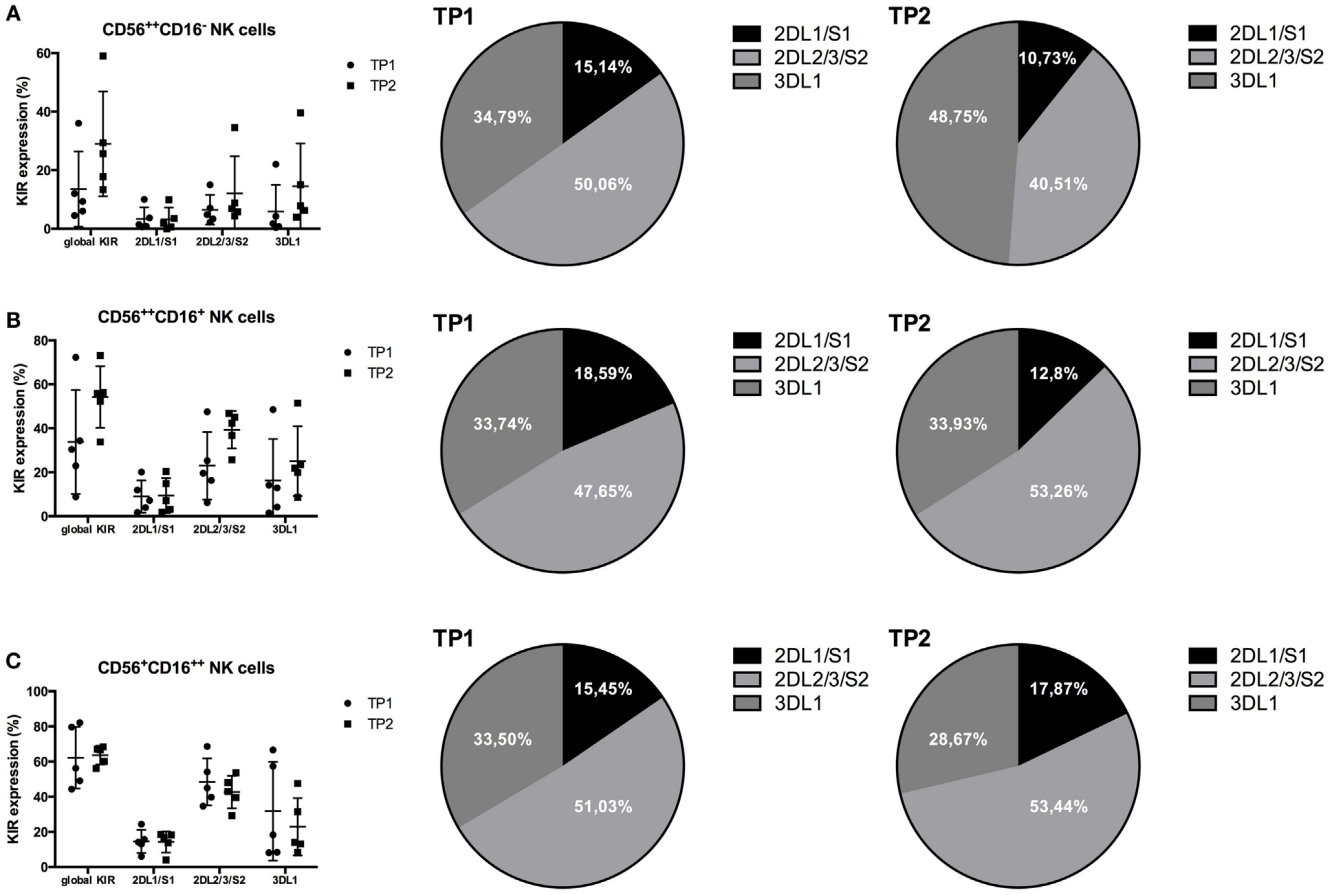
**(A)** Global KIR expression (anti-KIR2D and anti-KIR3DL1/2) was upregulated within the CD56^++^CD16^−^ NK cell population from TP1 to TP2 (TP1: 13.56%; TP2: 29.04%). KIR2DL1/S1 remained stable (TP1: 3.33%; TP2: 3.2%), whereas KIR2DL2/3/S2 (TP1: 6.49%; TP2: 12.08%) and KIR3DL1 (TP1: 5.85%; TP2: 14.54%) were both upregulated. **(B)** Within the CD56^++^CD16^+^ NK cells, the global KIR expression was upregulated from TP1 to TP2 (TP1: 33.74%; TP2: 54.24%) in addition to the KIR2DL2/3/S2 (TP1: 22.95%; TP2: 39.28%) and KIR3DL1 expression (TP1: 16.25%; TP2: 25.02%). In contrast, the KIR2DL1/S1 levels remained stable (TP1: 8.95%; TP2: 9.44%). **(C)** The expression levels of global KIR expression (TP1: 62.24%; TP2: 63.66%) as well as that of KIR2DL1/S1 (TP1: 14.65%; TP2: 14.26%) remained stable, whereas the two other KIR subsets revealed a slight decrease from TP1 to TP2 within the CD56^+^CD16^++^ NK cell population [2DL2/3/S2: 48.4% (TP1) and 42.65% (TP2); 3DL1: 31.77% (TP1) and 22.88% (TP2)]. The proportions of the different KIR subsets within the whole KIR population are indicated within the pie charts **(A–C)**.

We next performed an extended KIR analysis within the samples of five additional patients, who were not included into the original analysis group because we did not have a sample from TP3. We analyzed the expression levels and distribution of global KIR expression (anti-KIR2D and anti-KIR3DL1/2), as well as KIR2DL1/S1, KIR2DL2/3/S2, and KIR3DL1, within the different subsets at TP1 and TP2. Notably, global KIR expression levels were upregulated within both CD56^++^ NK cell populations at TP2 compared to TP1, whereas they remained stable within the CD56^+^CD16^++^ NK cell population, confirming the results from the former analyzed patient cohort (Figures 5A–C). Within the CD56^+^CD16^++^ population, no clear differences between the expression levels of the different KIR subsets were observed comparing TP1 and TP2 (Figure 5C). In contrast, both CD56^++^ NK cell subsets upregulated their KIR2DL2/3/S2 and KIR3DL1 expression levels from TP1 to TP2, whereas the KIR2DL1/S1 levels remained stable between the two time points (Figures 5A,B). Moreover, the proportion of the different KIR subsets within the global KIR population changed within the CD56^++^CD16^−^ population, with KIR2DL2/3/S2 being the dominant KIR subset at TP1 and KIR3DL1 being the dominant one at TP2 (Figure 5A; pie chart). Within the CD56^++^CD16^+^ population, KIR2DL2/3/S2 was the dominant KIR population at TP1 and was the only one to increase from TP1 to TP2 (Figure 5B; pie chart).

### NK Cell Function is Preserved After Leukocyte Recovery

Finally, we analyzed the functions of the different NK cell subsets at the three time points before and after HDC/autoSCT. After an overnight incubation with low-dose IL-2 (100 IU/ml) and 4 h of coculture with K562 cells, we investigated the cytokine (IFN-γ) and chemokine (MIP-1β) productions, as well as the NK cell degranulation (CD107a expression) capacity (for gating strategy see Figure S5 in Supplementary Material). Due to very low cell numbers at TP2, functional analysis was only possible in a subset of all included patients (*n* = 17).

**Figure 6:**
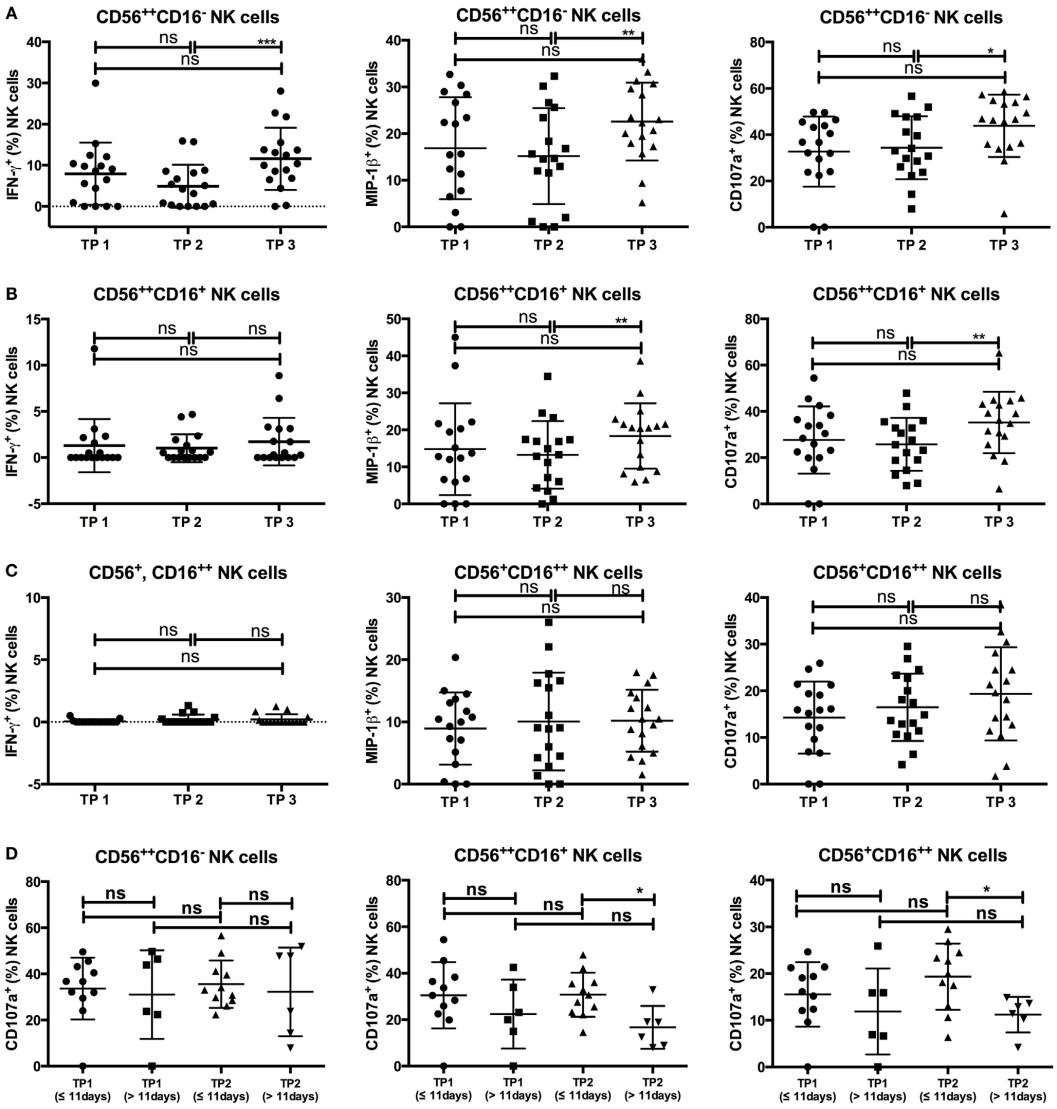
**(A)** The CD56^++^CD16^−^ subset revealed an increase of IFN-γ-, MIP-1β-, and CD107a-positive cells between TP2 and TP3 [TP2: 4.9% (IFN-γ), 15.17% (MIP-1β), 34.37% (CD107a), TP3: 11.58% (IFN-γ), 22.58% (MIP-1β), 43.82% (CD107a)], while the percentages between TP1 and TP2 were similar [TP1: 7.92% (IFN-γ), 16.89% (MIP-1β), and 32.7% (CD107a)]. **(B)** IFN-γ-positive cells within the CD56^++^CD16^+^ population were only marginal at all three time points (TP1: 1.29%; TP2: 1.01%; TP3: 1.72%), whereas MIP-1β- and CD107a-positive cells increased from TP2 to TP3 [TP2: 13.26% (MIP-1β), 25.78% (CD107a); TP3: 18.37% (MIP-1β), 35.26% (CD107a)] but not from TP1 to TP2 [TP1: 14.79% (MIP-1β), 27.64% (CD107a)]. **(C)** No IFN-γ-positive cells were detectable within the CD56^+^CD16^++^ population at any of the three time points. MIP-1β-positive cells remained stable throughout all three time points (TP1: 8.93%; TP2: 10.06%; TP3: 10.18%) which is similar to CD107a-expressing cells (TP1: 14.26%; TP2: 16.47%; TP3: 19.33%). **(D)** Patients were grouped according to the duration between SCT and TP2 (≤11 vs. >11 days). While there were no differences between the percentage of CD107a-expressing cells within the CD56^++^CD16^−^ population at TP1 and TP2, the percentage of CD107a-positive NK cells at TP2 was lower within patients with a longer duration between SCT and TP2 within the CD56^++^CD16^+^ (≤11 days: 30.74%; >11 days: 16.68%) and the CD56^+^CD16^++^ (≤11 days: 19.34%; >11 days: 11.21%) populations.

As expected, CD56^++^CD16^−^ NK cells were the main subset to produce IFN-γ upon interaction with K562 cells at all three time points (Figure 6A). The percentage of IFN-γ-positive CD56^++^CD16^−^ NK cells was slightly decreased at TP2 compared to TP1 but significantly increased from TP2 to TP3 (*p*-value: 0.0008). Similarly, MIP-1β- and CD107a-positive CD56^++^CD16^−^ cells remained constant between TP1 and TP2, whereas their percentages increased from TP2 to TP3 [*p*-values: 0.0056 (MIP-1β) and 0.0232 (CD107a)].

Whereas IFN-γ production was only marginal at all three time points within the CD56^++^CD16^+^ NK cell population (Figure 6B), MIP-1β- and CD107a-positive cells had similar percentages at TP1 and TP2. Both percentages significantly increased from TP2 to TP3 (*p*-value: 0.0079 for both).

Within the CD56^+^CD16^++^ NK cell subsets, the percentage of MIP-1β- and CD107a-positive NK cells after coincubation with K562 cells remained constant at all three time points, whereas no IFN-γ-positive NK cells were detected at any time point (Figure 6C).

Although the number of available patient samples was low, we tried to correlate the NK cell function values with clinical data. Remarkably, there was an impact of the duration from SCT to TP2 (≤11 vs. >11 days), as the percentage of CD107a-positive cells within the CD56^++^CD16^+^ and CD56^+^CD16^++^ populations was significantly lower when the time period between SCT and TP2 exceeded 11 days [*p*-values: 0.0111 (CD56^++^CD16^+^); 0.027 (CD56^+^CD16^++^); Figure 6D]. In investigating age-dependent differences (≤56 vs. >56 years), we observed that patients older than 56 years tended to have slightly higher percentages of IFN-γ- and MIP-1β-positive CD56^++^CD16^−^ cells at TP1, although the difference was not significant. Furthermore, no significant differences in the presence of MIP-1β- or CD107a-positive CD56^++^CD16^+^ or CD56^+^CD16^++^ NK cells between the two age groups were observed at all three time points, although older patients tended to have lower CD107a expression within the CD56^+^CD16^++^ subset at TP3 (*p*-value: 0.074). Notably, the observed increase of CD107a-positive CD56^+^CD16^++^ cells from TP2 to TP3 was only present in younger but not older patients [≤56 years: 15.69% (TP2) and 24.05% (TP3), *p*-value: 0.1; >56 years: 17.16% (TP2) and 15.14% (TP3), p-value: 0.82; Figure S6 in Supplementary Material].

## Discussion

In the setting of HDC/autoSCT, it has been demonstrated that a rapid NK cell recovery at 1 month after HDC/autoSCT is associated with a prolonged progression-free survival in MM (23) and NHL patients (16). In those studies, the absolute NK cell count (cells/μl) at 1 month or 15 days after HDC/autoSCT was investigated, whereas in our study, we analyzed the NK cell percentage within the leukocyte population in correlation with the day of leukocyte recovery following autoSCT.

Our data demonstrate that the percentage of NK cells within the leukocyte population decreased after leukocyte recovery but increased to the initial levels over time. Notably, when the time period between SCT and TP2 was >11 days, indicating a delay in leukocyte recovery, the decrease within the NK cell percentage was lower. This may be explained by the fact that leukocyte recovery (white blood cell count >1000/μl) after SCT is mainly due to the recovery of neutrophil granulocytes (10), and their recovery can be delayed in contrast to NK cell recovery (24). Moreover, the higher increase of the NK cell percentage from TP2 to TP3 in patients from whom the third blood sample was collected ≤38 days after TP2 indicates that the NK cell percentage within the leukocytes increases much more within the first month after leukocyte recovery and then decreases again. Similar results have been demonstrated by Rueff et al. by analyzing the absolute NK cell count numbers (cells/μl) 1, 3, 6, 12, and 24 months after SCT. Here, the NK cell numbers first increased 1 month after SCT, but then they decreased again until 6 months after SCT (23). The absence of a decrease in the NK cell percentage at TP2 within patients with recurrent/refractory disease at 1 year after SCT could be explained by the fact that most of these patients (4/6; 66.6%) had a time period of >11 days from SCT to TP2, unlike non-relapsing/refractory patients (8/21; 38%).

In line with other NK cell reconstitution studies after SCT (24–26), we observed elevated percentages of the more immature CD56^++^CD16^+/−^ NK cell subsets shortly after SCT, decreasing only slowly at later time points. As the ratio between CD56^+^/CD56^++^ NK cells did not differ with shorter (≤38 days) or longer (>38 days) time periods between TP2 and TP3, we assume that normalization of the NK cell subset distribution takes much longer than the recovery of the NK cell numbers. Similar observations have been made within patients receiving an allogeneic SCT after reduced-intensity conditioning (26). The conditioning and former treatment regimens could explain the different NKG2A^+^ NK cells ratios between myeloma and lymphoma patients at TP2.

Moreover, the percentage of NKG2A^+^ NK cells was increased after HDC/autoSCT and remained high even after several months, as it has been recently demonstrated by Pical-Izard et al. after allogeneic SCT (26). In contrast, we could demonstrate a highly significant increase of CD57^+^ and KIR^+^ NK cells, mainly within the CD56^++^CD16^+/−^ subsets at TP2. This effect has not been described thus far within the literature because most of the studies have evaluated NK cell subsets 1 month after SCT (23–27). At this time point, the percentage of CD57^+^ and KIR^+^ NK cells had already decreased back toward normal levels within our study group. The prolonged immature phenotype in the Pical-Izard study may be attributed to GVHD prophylaxis, especially cyclosporine A (CSA). Vukicevic et al. investigate the NK cell phenotype at an equally early time point after allogeneic stem cell transplantation as we did but did not detect an upregulation of KIRs within the CD56^++^ subsets (28), which might be due to the allogeneic transplantation setting. Acquisition of CD57 and KIRs as well as downregulation of NKG2A has been demonstrated as signs of NK cell differentiation and maturation (22). It is known that CD57 expression increases with age within the CD56^+^CD16^++^ population (29), whereas we did not observe any age-related differences within this NK cell subset at any time point. Nevertheless, an age-dependent difference was observed at TP2 within the CD56^++^CD16^−^ population.

Therefore, the question arises whether these CD56^++^ cells described here represent more mature new NK cells or are just activated “old” NK cells that might have increased their CD56 expression and lost CD16 on their surface. As this phenotype is reset after at least 2 weeks after leukocyte regeneration, one could argue that the phenotype shift is due to the cytokine milieu during HDC/autoSCT. Indeed, there have been several reports about increased cytokine concentrations during allogeneic and autologous SCT (16, 25, 30) shaping the NK cell phenotype. It has been reported that CD56^+^CD16^++^ NK cells are capable of up-regulating CD56 expression upon IL-15 (28) or IL-12 stimulation (31), of which IL-15 is known to be increased during HDC/autoSCT (16). Furthermore, different groups demonstrated a downregulation of CD16 by metalloproteinases, which can be induced by IL-2 (32, 33). In general, the combined effect of CD56 upregulation upon IL-15 stimulation and the loss of CD16 through IL-2-stimulated upregulation of metalloproteinases might result in the observed CD56^++^CD16^+/−^ NK cell phenotype at TP2. Nevertheless, the CD56^++^ NK cells described in our study upregulated KIR3DL1 and KIR2DL2/3/S2, while KIR2DL1/S1 remained stable. KIR3DL1 and KIR2DL2/3 are the first KIRs expressed after SCT, whereas KIR2DL1 is upregulated quite late (34, 35). Therefore, we could assume that the KIR upregulation was due to the generation of “fresh/new” NK cells and was not due to a shift of “old” NK cells to a CD56^++^ phenotype because we should have observed no changes within the KIR subtype distribution between TP1 and TP2 if the cells were derived from the “old” NK population. Moreover, we observed a slight upregulation of CX3CR1 at TP2 (data not shown). CX3CR1 expression is associated with a more mature and differentiated NK cell phenotype within healthy donors (36). In contrast to the observed CD56 upregulation, which can be explained by IL-15 stimulation (28), CX3CR1 is known to be downregulated upon IL-15 stimulation (36), which contradicts the idea that CD56^++^ NK cells with a mature phenotype (CD57^+^, CX3CR1^+^, and KIR^+^) have arisen from CD56^dim^ NK cells. In future studies, it would be very interesting to investigate which factors are responsible for this NK cell phenotype because protocols for inducing NK cell maturation and differentiation have yet to be optimized.

Most importantly, we analyzed NK cell functional activity directly after leukocyte recovery after HDC/autoSCT. Upon interaction with K562 tumor cells, the percentage of IFN-γ- and MIP-1β-positive CD56^++^CD16^+/−^ NK cells did not differ between TP1 and TP2. This result demonstrates that NK cells are capable of recognizing tumor cells and inducing cytokine and chemokine production, even at a very early time point after HDC/autoSCT. Moreover, the degranulation capacity of the CD56^+^CD16^++^ NK cell subset, known to be mainly responsible for NK cell cytotoxicity (37), remained stable throughout the whole time period until TP3, indicating that these NK cells were able to kill tumor cells at an early time point after SCT. This finding is consistent with other studies in which patients received allogeneic SCT. For example, in the setting of HLA-matched SCT after reduced-intensity conditioning, it has been demonstrated that the NK cell degranulation and chemokine production capacity was similar to healthy controls as early as 1 month after SCT. In contrast to our data, IFN-γ production upon interaction with K562 cells was significantly reduced after SCT compared to healthy donors (26). These differences might be due to the use of immunosuppressive drugs such as CSA because it has been demonstrated that CSA is able to reduce IFN-γ production upon target-cell interaction (38), although a recent report has failed to demonstrate such an effect (39). Furthermore, we compared IFN-γ production upon interaction with K562 cells before and after SCT and not directly with healthy control samples. Therefore, although we did not observe a significant decrease in IFN-γ-positive cells between TP1 and TP2, their percentage might be still significantly lower than in healthy controls. Additionally, we observed that the degranulation capacity was influenced by the time period between SCT and TP2 because patients with a time period >11 days had significantly reduced CD107a-positive CD56^+^CD16^++^ cells at TP2. Because the prolonged time from SCT to TP2 indicates a longer period for leukocyte recovery, this might give an explanation for the reduced degranulation capacity. Nevertheless, we could not discover a correlation between NK cell function at TP2 and the rate of recurrent/refractory disease at 1 year after SCT, which might be due to the low number of recurrent/refractory patients and the short follow-up period.

To the best of our knowledge, this is the first study investigating NK cell function at such an early time point after HDC/autoSCT. We were able to demonstrate that NK cells were capable of cytokine/chemokine production and degranulation upon tumor cell interaction. Furthermore, we describe an unusual CD56^++^ NK cell population expressing high levels of CD57 and KIRs shortly after SCT. Further analysis and characterization of this population might reveal more details about how NK cell maturation and differentiation are regulated.

## Author Contributions

All authors critically revised the work for important intellectual content and approved the final version of the manuscript. They all agreed to be accountable for all aspects of the work in ensuring that questions related to the accuracy or integrity of any part of the work are appropriately investigated and resolved. BJ, ST, and KP were responsible for acquisition, analysis, and interpretation of the data. EU, PB, AM, and BJ were responsible for the concept and design of the work.

## Conflict of Interest Statement

The authors declare that the research was conducted in the absence of any commercial or financial relationships that could be construed as a potential conflict of interest.

## Abbreviations

HDC, high-dose chemotherapy; (auto)SCT, (autologous) stem cell transplantation; MM, multiple myeloma; NHL, non-Hodgkin lymphoma; TP1–3, time point 1–3; KIRs, killer Ig-like receptors; NK cell, natural killer cell.
