# Supplementary material for: NK Cell Subgroups, Phenotype, and Functions After Autologous Stem Cell Transplantation
Source: Front Immunol. 2015 Nov 24;6:583. doi: 10.3389/fimmu.2015.00583 (PMC4657185; doi:10.3389/fimmu.2015.00583)
Supplement: Supplementary file 1 [file Data_Sheet_1.PDF]

Supplementary Material

NK cell subgroups, phenotype and functions after autologous stem cell transplantation

Benedikt Jacobs[1,2,3], Sara Tognarelli[4,5], Kerstin Poller[3], Peter Bader[4,5], Andreas Mackensen[3], Evelyn Ullrich[3,4,5*]

[1]Department of Cancer Immunology, Institute for Cancer Research, Oslo University Hospital, Radiumhospital, Oslo, Norway

[2]The KG Jebsen Center for Cancer Immunotherapy, Institute of Clinical Medicine, University of Oslo, Oslo, Norway,

[3]Department of Haematology and Oncology, University Hospital Erlangen, Erlangen, Germany

[4]Children's Hospital, Department of Pediatric Stem Cell Transplantation and Immunology, Johann Wolfgang Goethe-University, Frankfurt, Germany

[5]LOEWE Center for Cell and Gene Therapy, Johann Wolfgang Goethe-University, Frankfurt, Germany

*** Correspondence:** Prof. Dr. Evelyn Ullrich, Children's Hospital, Johann Wolfgang Goethe-University, Theodor-Stern-Kai 7, House 32B, 60596 Frankfurt, Germany

evelyn.ullrich@kgu.de

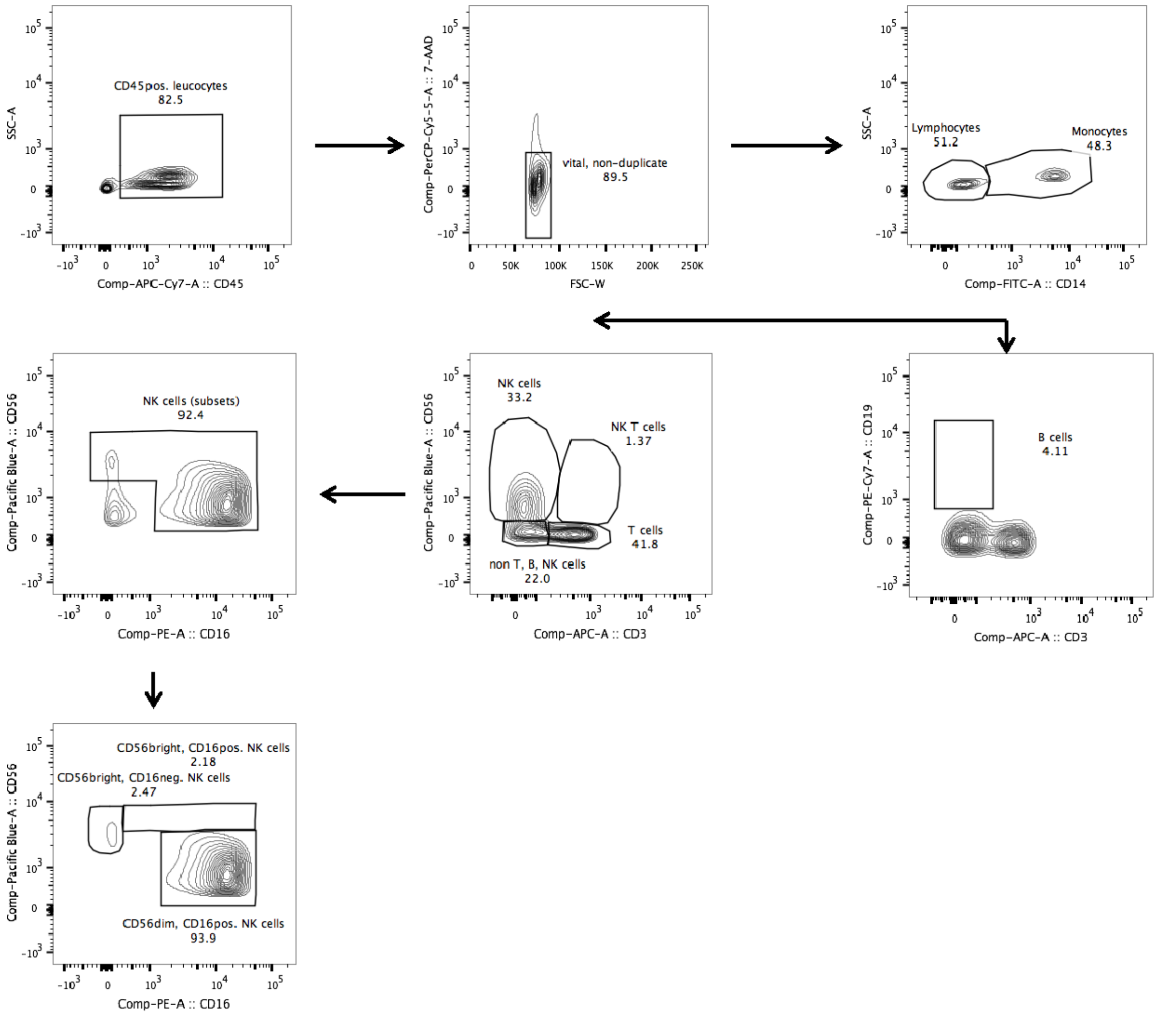

Supplementary Figure S1. Gating strategy for the evaluation of different leukocyte subsets and their percentages within the whole lymphocyte population. Percentages of the different subsets were calculated in relation to CD45-positive, 7-AAD-negative, non-duplicate cells, defined as lymphocytes. After gating on CD56-positive and CD3-negative NK cells, the CD56-low, CD16-negative NK cells were excluded prior to further analyses of the different NK cell subsets because it has been reported that this population can result due to the thawing process of the PBMC samples (*Lugthart G.; Blood 2015*).

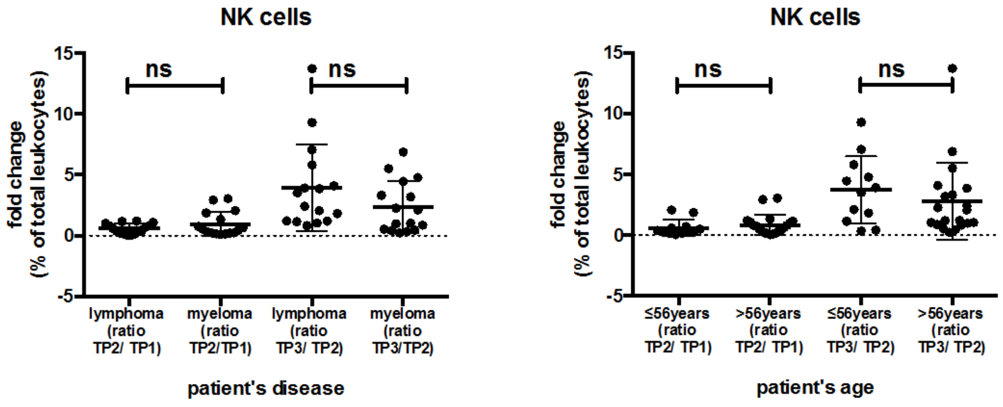

Supplementary Figure S2. The fold change in the NK cell percentage within the leukocyte population at defined time points did not differ between the patient groups depending on patients' malignant disease (p-value TP2/TP1: 0.533; p-value TP2/TP1: 0.0858) or age (p-value TP2/TP1: 0.25; p-value TP2/TP1: 0.223).

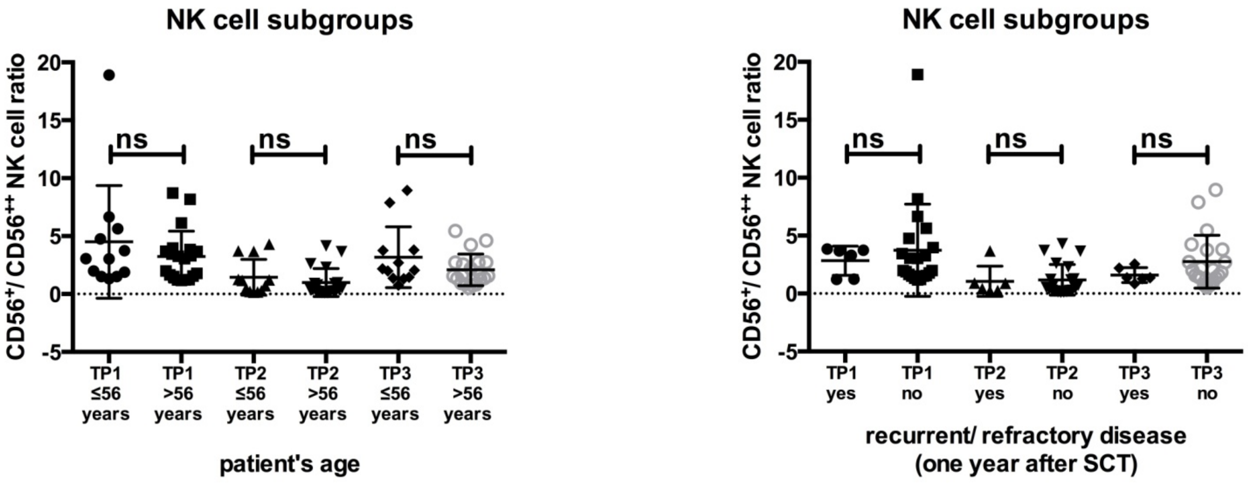

Supplementary Figure S3. The $CD56^{+}$/ $CD56^{++}$ ratio did not differ at the three defined time points when comparing the patients' age (≤56 years vs. >56 years) or relapse/ refractory disease one year after SCT (yes vs. no).

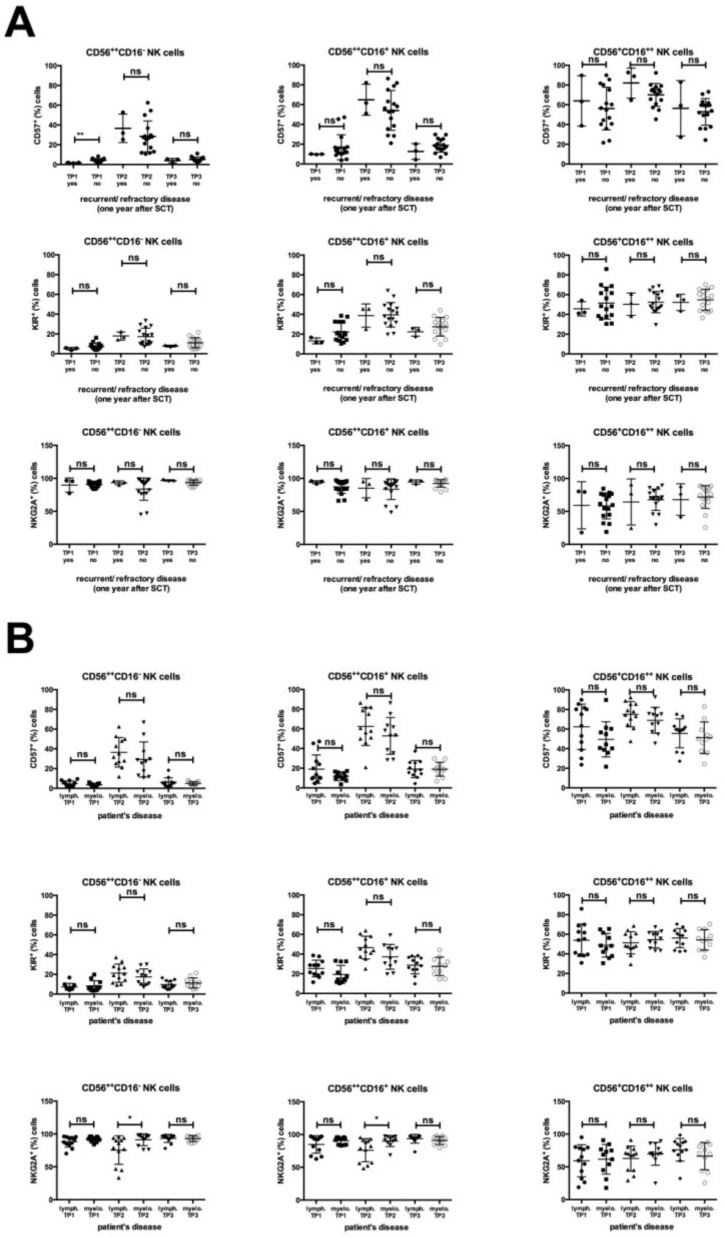

Supplementary Figure S4. The CD57, KIR and NKG2A expression within the three major NK cell subsets at the three different time points were analyzed depending on whether the patients were either **(A)** recurrent or refractory one year after SCT (yes) or not (no) or whether they **(B)** had a lymphoma or myeloma as their hematological malignancy. The only differences were observed in the CD57 expression within the CD56^{++}CD16^{-} NK cell population at TP1 when comparing patients having refractory/ recurrent disease one year after SCT (1.783%) to those that did not (4.219%; p-value: 0.0083). Moreover, the NKG2A expression within the CD56^{++}CD16^{+}/$^{-}$ NK cells at TP2 was significantly lower in lymphoma (CD16^{-}: 75.5%; CD16^{+}: 75.98%) than in myeloma patients (CD16^{-}: 91.13%; CD16^{+}: 90.11%; p-value: 0.0163 (CD16^{-}), 0.0196 (CD16^{+})).

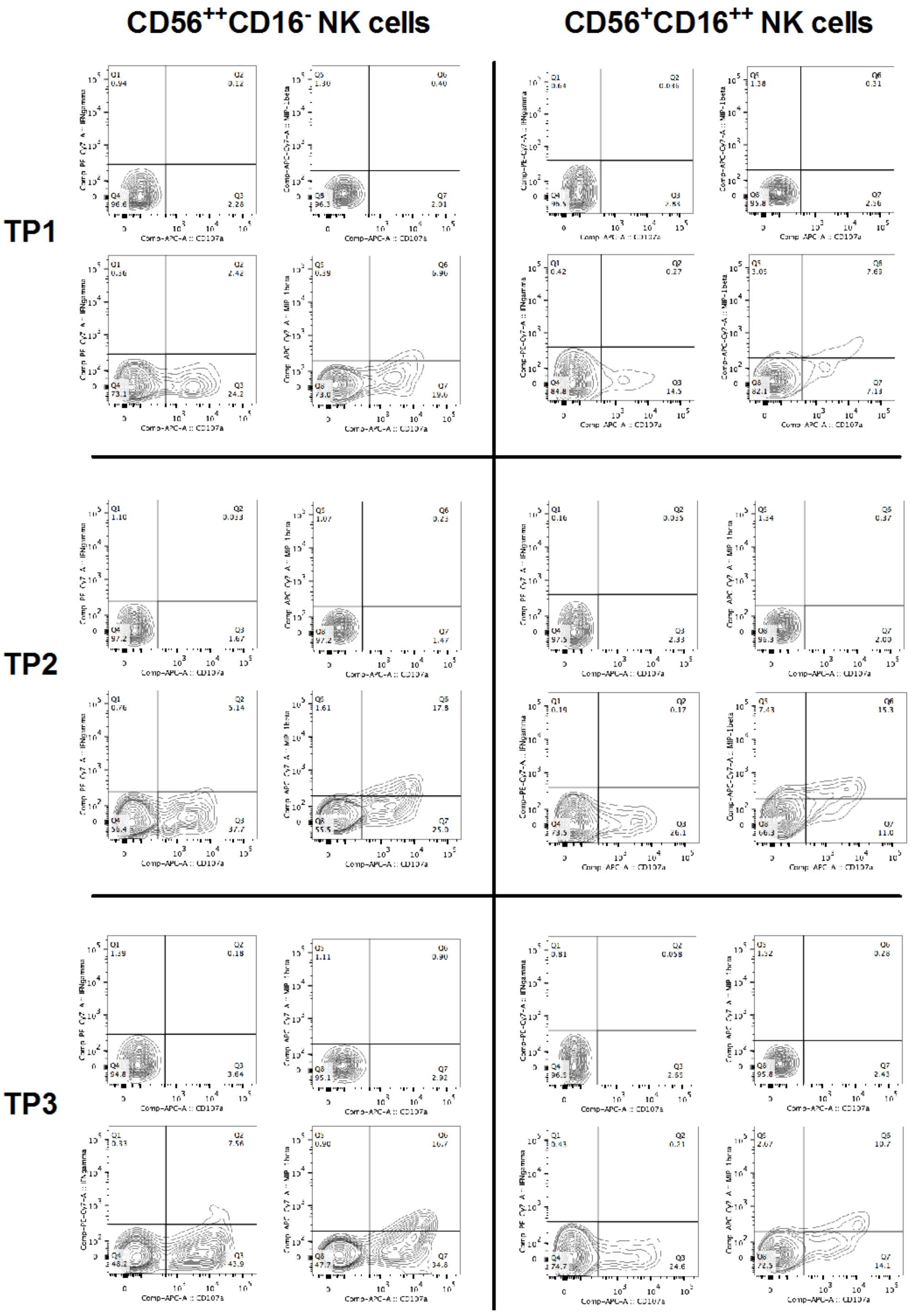

Supplementary Figure S5. Representative FACS plots showing degranulation, IFNγ- and MIP-1β production upon contact with K562 cells within the CD56++CD16- and CD56+CD16++ NK cell subsets of one donor. At the indicated time points (TP1-3) FACS plots in the upper row represent NK cell functions without and in the lower row together with K562 cells. Gates were set according to the negative population within the NK cell population without K562 cells.

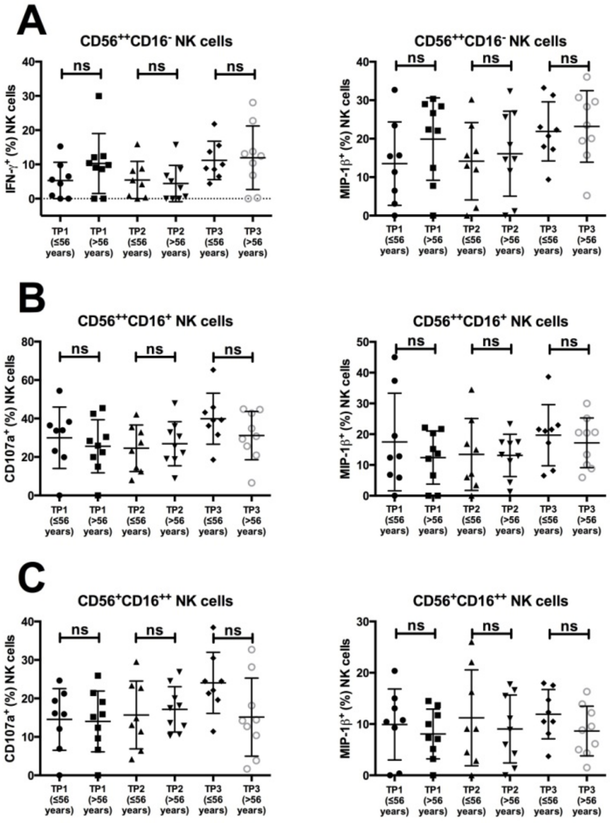

Supplementary Figure S6. (A) The percentage of IFN-γ- and MIP-1β–positive $CD56^{++}CD16^{-}$ cells at TP2 (IFN-γ: 5.44% (≤56 years), 4.41% (>56 years); MIP-1β: 14.14% (≤56 years), 16.1% (>56 years)) and TP3 (IFNγ: 11.16% (≤56 years), 11.95% (>56 years); MIP-1β: 21.89% (≤56 years), 23.18% (>56 years)) did not differ significantly between patients older or younger than 56 years. Older patients tended to have slightly higher levels at TP1 (IFN-γ: 5.29% (≤56 years), 10.26% (>56 years); MIP-1β: 13.52% (≤56 years), 19.89% (>56 years)). **(B-C)** No significant differences were observed between the percentage of MIP-1β- or CD107a-positive $CD56^{++}CD16^{+}$ or $CD56^{+}CD16^{++}$ NK cells in relation to patients age at any of the three time points. Nevertheless, older patients tended to have lower CD107a expression levels within the $CD56^{+}CD16^{++}$ subset at TP3 (<56 years: 24.05%; >56 years: 15.14%).
